# Intrapartum Coccygeal Fracture in a Young Female: A Case of Prolonged Postpartum Coccygodynia

**DOI:** 10.7759/cureus.77233

**Published:** 2025-01-10

**Authors:** Laila Alhubaishi, Raya Flayyih, Heba Adan, Amany M Altabba, Rawan Flayyih, Mohamad M Assker

**Affiliations:** 1 Obstetrics and Gynaecology, Latifa Hospital, Dubai, ARE; 2 Education, Dubai Health, Dubai, ARE; 3 Obstetrics, University of Sharjah, Sharjah, ARE; 4 Medicine, University of Sharjah, Sharjah, ARE; 5 Radiology, Sheikh Khalifa Medical City, Abu Dhabi, ARE

**Keywords:** coccygeal fracture, coccyx pain, normal vaginal delivery, pregnancy, risk factors

## Abstract

Intrapartum coccygeal fractures are rare but significant injuries occurring during childbirth. The coccyx, or tailbone, is vulnerable to trauma during labor, especially in difficult or instrument-assisted deliveries. The exact incidence is unclear due to underreporting and misdiagnosis, often mistaken for common postpartum discomforts. Risk factors include macrosomia, instrument-assisted deliveries, prolonged labor, previous pelvic trauma, and variations in maternal pelvic anatomy. Clinical presentation typically involves localized pain, swelling, tenderness, bruising, and difficulty in movement. Diagnosis is based on clinical evaluation and imaging, with MRI being particularly useful. Management is primarily conservative, focusing on pain relief, physical therapy, and activity modification. In persistent cases, surgical intervention may be necessary. Most patients recover well, though some may experience prolonged discomfort.

We report a rare case of a 31-year-old p1+0 experiencing severe lower back pain post-vaginal delivery that was reassured in the postpartum visit as inflammation and coccydynia. However, despite initial conservative management for presumed inflammation/coccydynia, the pain persisted, severely affecting her daily activities and professional life as a dental hygienist. After one and a half year, she was seen by an orthopedic surgeon, and thorough detailed history, physical examination, and MRI confirmed a transverse fracture of the second coccygeal segment. Accordingly, the patient was counselled and started on conservative treatments including analgesia and physiotherapy sessions, which provided limited relief. Therefore, the patient was recommended for surgical intervention (excision of the coccyx), but she refused. She sought alternative treatments abroad, receiving shock wave therapy and caudal block injections, which successfully controlled her pain. This case underscores the importance of prompt diagnosis and personalized management strategies for intrapartum coccygeal fractures to optimize patient outcomes.

## Introduction

Low-back and buttock pain is a common complaint during pregnancy and the postpartum period, and it may be due to a variety of conditions. However, intrapartum coccygeal fracture is a relatively rare but significant complication that can occur during childbirth [1]. The coccyx, or tailbone, is the terminal segment of the vertebral column and plays a crucial role in maintaining stability and support in the seated position.

During vaginal delivery, particularly in cases involving prolonged labor, large fetal size (macrosomia), maternal pelvic anatomy, or instrumental delivery [2]. The coccyx can be subjected to excessive pressure and trauma, leading to fracture. This condition often results in severe pain localized to the tailbone, especially with sitting or pressure. Additionally, symptoms may include swelling and tenderness around the coccyx region, bruising of the overlying skin, and difficulty in movement [3]. Ultimately, this can lead to functional impairment for the affected individual, necessitating prompt recognition and appropriate management to alleviate pain and support healing.

Diagnosing a coccygeal fracture typically involves a combination of clinical evaluation and imaging. A detailed history and physical examination focusing on the localization of pain and tenderness can suggest a coccygeal injury. Radiographs or MRI can confirm the diagnosis, with MRI being particularly useful for identifying soft tissue involvement and providing a detailed assessment of the coccygeal area. Patel et al. highlighted that X-ray imaging can often miss subtle fractures, whereas MRI offers a more accurate assessment of the coccygeal structure and associated soft tissue damage [4].

Management typically includes analgesia and non-steroidal anti-inflammatory drugs (NSAIDs), along with coadjuvant therapies such as rehabilitation, pelvic floor massage, infiltrations, psychotherapy, or, in severe cases, surgery [5]. In cases where conservative management fails and the patient experiences persistent pain and functional impairment, surgical intervention such as coccygectomy (removal of the coccyx) may be considered [6]. Most patients with coccygeal fractures recover with conservative management. However, some may experience chronic pain or discomfort, which can impact their quality of life. Long-term outcomes are generally favorable, though the recovery period can be prolonged, sometimes lasting several months.

To date, only two cases of intrapartum coccygeal fracture have been published in the literature. This case report aims to highlight the clinical presentation, diagnostic challenges, and therapeutic approaches associated with intrapartum coccygeal fracture, contributing to a better understanding of this under-recognized obstetric complication.

## Case presentation

A 31-year-old female, P1+0, delivered a healthy baby of 2.8 kg through IVF at 39 weeks of gestation in December 2021 via normal vaginal delivery with an episiotomy. Immediately following delivery, she experienced severe localized pain in her lower back (coccyx). During her postpartum follow-up visit, she was reassured, but due to the persistent severity of the pain, she sought evaluation at multiple hospitals. She was initially diagnosed with inflammation/coccydynia and began conservative management.

**Figure 1:**
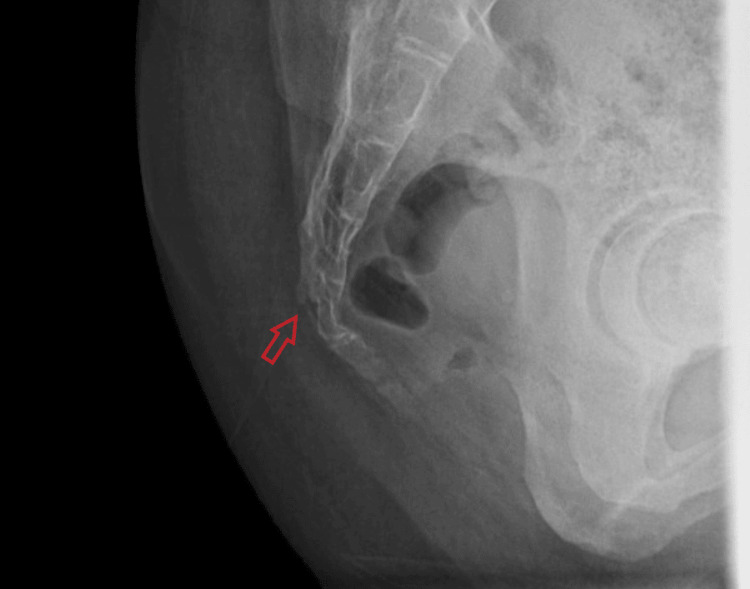
Lateral projection X-ray of the coccyx. Red arrow indicates transverse, complete, and anteriorly displaced fracture of the Cy2 segment of the coccyx.

After a year and a half, her condition began to significantly interfere with her daily life, particularly because she works as a dental hygienist and could not sit for more than three minutes. Consequently, she consulted an orthopedic surgeon in a private hospital for further evaluation and management. A detailed history and physical examination revealed localized tenderness over the coccyx, with a normal range of motion, normal gait, and a negative straight leg raise test. An initial X-ray was performed, which demonstrated a complete, transverse, and anteriorly displaced fracture of the Cy2 coccygeal segment (Figure 1).

**Figure 2:**
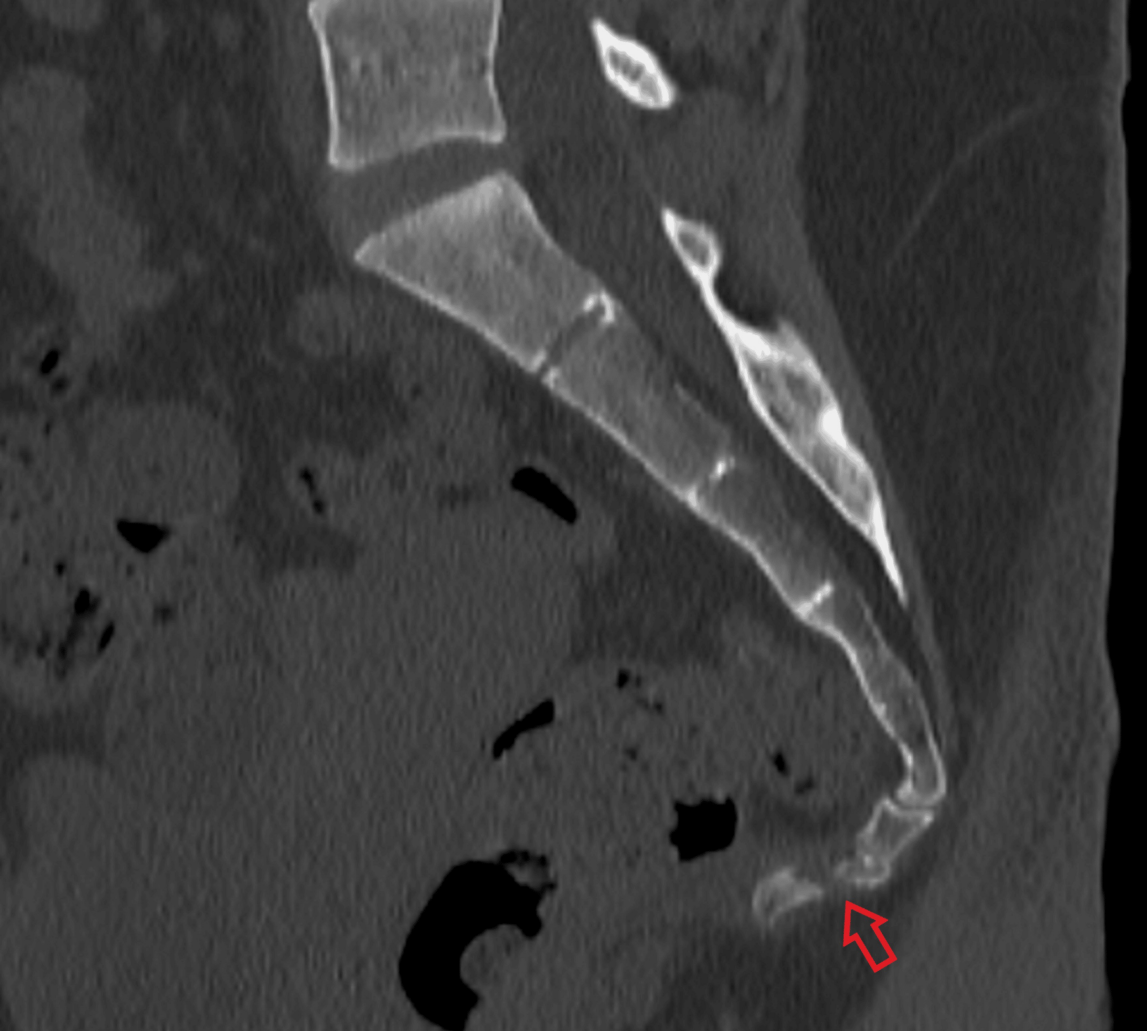
CT (sagittal view) of the coccyx Red arrow indicates transverse, complete, and anteriorly displaced fracture of the Cy2 segment of the coccyx, with traction measuring 3 mm.

A CT scan was then performed, redemonstrating fracture segments with potential healing process (Figure 2).

**Figure 3:**
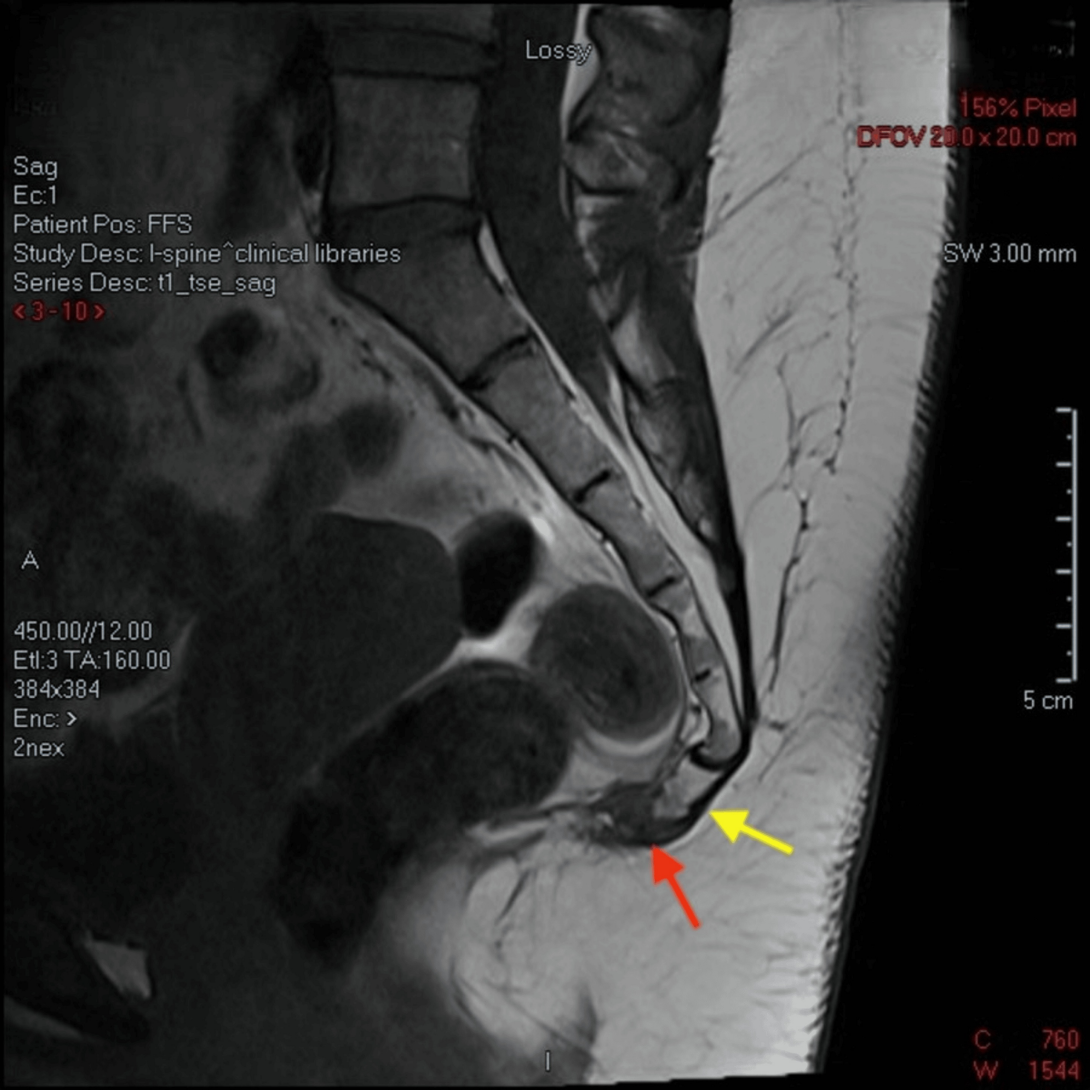
Non-contrast sagittal T1 MRI sequence Yellow arrow indicates transverse, complete, and anteriorly displaced fracture of the Cy2 segment of the coccyx, with fragment traction measuring 3 mm. Red arrow indicates a well-demarcated T1 hypointensity in the coccygeal synchondrosis, remarkable for early callous bone formation.

**Figure 4:**
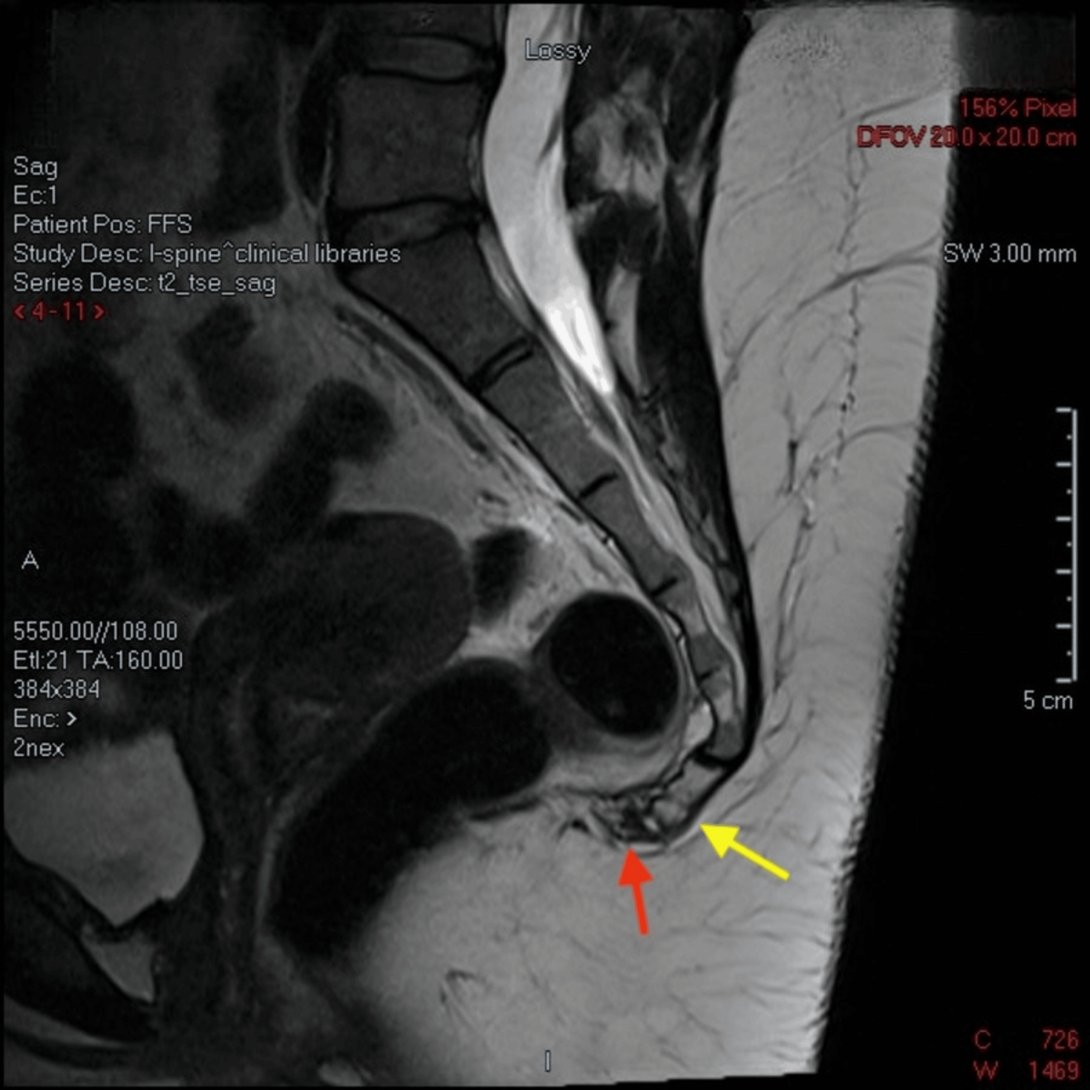
Non-contrast sagittal T2 MRI sequence Yellow arrow indicates transverse, complete, and anteriorly displaced fracture of the Cy2 segment of the coccyx, with fragment traction measuring 3 mm. Red arrow indicates well-demarcated T2 mixed hypo- and hyperintensity in the coccygeal synchondrosis remarkable for early callous bone formation.

Further imaging with MRI confirmed a fracture through the second coccygeal segment. Demonstrated changes shown on T1 (Figure 3) and T2 (Figure 4) MRI sequences are in keeping with callus formation.

She was counseled and advised to continue conservative management, including analgesia, the use of a donut seat cushion, and referral to a physiotherapy and rehabilitation center. She was very compliant with the sessions; however, despite completing the maximum number of sessions, she experienced only around 40% improvement in her symptoms. Therefore, she was advised for surgical intervention (excision of the coccyx), but she refused.

In August 2023, she traveled to Thailand for further management and received a single session of shock wave therapy. Currently, her pain is controlled with caudal block injections.

## Discussion

Even in cases in which pregnancy is considered uncomplicated, there is still a significant chance of unexpected complications during labor [7]. Musculoskeletal problems may occur during the peripartum period and lead to significant impairment in women's lives [8]. Coccydynia (discomfort in the tailbone) is a common complication observed after vaginal delivery [9]. While coccygeal fractures and luxations are rare, they should still be considered alongside ligament strains or sprains as potential causes [2]. A review of the literature revealed only three reported cases of intrapartum coccygeal fractures, one of which was presented in the form of poster [1,2,10]. As noted by Foye et al., coccyx fractures and dislocations can be challenging to diagnose and treat [11]. This was evident in our case, where the correct diagnosis was made only after a year and a half. Coccygeal fractures have nonspecific signs and symptoms [1]. However, chronic lower back pain and pain while sitting were common among all the cases. Dyschezia and fecal urgency may also be seen [10], which was the reason the patient was referred to the proctology clinic in our case. To confirm the diagnosis of coccygeal fracture, lateral radiography of the coccyx may be performed to check for any misalignment [10]. In the published cases, CT has a role in diagnosing the fracture. MRI is helpful in detecting soft tissue involvement and is the preferred method for closely examining the coccyx area [4]. Notably, other rare intrapartum fractures, such as sacral fractures, have been reported and are associated with transient osteoporosis related to pregnancy. Therefore, evaluation of calcium, phosphate, and vitamin D levels is recommended [12], which was missed in this case. Other risk factors could be maternal pelvic anatomy, prolonged labor, and macrosomia [3].

The treatment is mostly conservative, including NSAIDs, sitting on a donut-shaped pillow, using ice, and physical therapy [10]. However, in our case, it aggravated the pain, especially the shockwave therapy. It is essential to make the diagnosis as early as possible and start the appropriate treatment to prevent complications such as introital dyspareunia and pelvic floor myalgia [3]. In the literature, the diagnosis was made within months or weeks, which is considered early, and yielded positive outcomes in patients. However, in our case, since the diagnosis was made after a year and a half, the pain did not subside and, in fact, became debilitating. A clinical trial conducted in Spain reported that ganglion impar block and peridural caudal blocking may be highly effective in cases with hard-to-control coccydynia [13]. The caudal block was performed on our patient, resulting in a reduction in her pain. Recent studies recommend reserving surgical options for refractory cases, as the risk of major complications is high [14]. It was an option recommended for our patient, but she chose to decline it. Identifying rare occurrences such as coccygeal fractures is crucial as it enhances clinical judgment and helps doctors consider these diagnoses earlier, sparing patients’ time and pain.

## Conclusions

Coccygeal fractures during labor are rare but important to consider in the early diagnosis of postpartum coccydynia. The coccyx, supported by sacrococcygeal ligaments, elongates during labor to provide space for the fetus, making it vulnerable to injury. These fractures often go undetected due to nonspecific symptoms but may be indicated by localized pain, especially when sitting on hard surfaces or changing posture. Undiagnosed coccygeal fractures can contribute to chronic pain conditions, such as fibromyalgia, and may remain unnoticed for extended periods. During labor, posterior dislocation of the coccyx is common due to the pressure from the fetal head, while anterior subluxation may present as persistent buttock pain with localized tenderness. Early recognition and proper diagnosis are crucial to managing these injuries effectively.
